# An Attempt to Optimize Supercritical CO_2_ Polyaniline-Polycaprolactone Foaming Processes to Produce Tissue Engineering Scaffolds

**DOI:** 10.3390/polym14030488

**Published:** 2022-01-26

**Authors:** Antonio Montes, Diego Valor, Laura Delgado, Clara Pereyra, Enrique Martínez de la Ossa

**Affiliations:** Department of Chemical Engineering and Food Technology, Faculty of Sciences, University of Cadiz, International Excellence Agrifood Campus (CeiA3), Campus Universitario Río San Pedro, 11510 Puerto Real, Cadiz, Spain; diego.valor@uca.es (D.V.); lauradelgadogallego@gmail.com (L.D.); clara.pereyra@uca.es (C.P.); enrique.martinezdelaossa@uca.es (E.M.d.l.O.)

**Keywords:** polycaprolactone, polyaniline, supercritical CO_2_ foaming, scaffolds, conjugated polymers

## Abstract

Conjugated polymers are biomaterials with high conductivity characteristics because of their molecular composition. However, they are too rigid and brittle for medical applications and therefore need to be combined with non-conductive polymers to overcome or lessen these drawbacks. This work has, consequently, focused on the development of three-dimensional scaffolds where conductive and non-conductive polymers have been produced by combining polycaprolactone (PCL) and polyaniline (PANI) by means of supercritical CO_2_ foaming techniques. To evaluate their therapeutic potential as implants, a series of experiments have been designed to determine the most influential variables in the production of the three-dimensional scaffolds, including temperature, pressure, polymer ratio and depressurization rate. Internal morphology, porosity, expansion factor, PANI loads, biodegradability, mechanical and electrical properties have been taken as the response variables. The results revealed a strong influence from all the input variables studied, as well as from their interactions. The best operating conditions tested were 70 °C, 100 bar, a ratio of 5:1 (PCL:PANI), a depressurization rate of 20 bar/min and a contact time of 1 h.

## 1. Introduction

The use of biocompatible materials as implants is currently the focus of many research projects in the area of tissue engineering. These biomaterials would avoid adverse immunologic reactions and provide a protective and supportive means for self-sufficient cells.

Most implantable biomedical devices used, from coronary vascular stents to bone implants, are often made up of ceramics, metals and polymers. Ceramics offer presumed good corrosion resistance and high biocompatibility for bone implants, while metals have good strength and wear resistance to satisfy the mechanical requirements of orthopedic implants [1]. However, the use of these implants have disadvantages such as the high brittleness and difficulty in manufacturing of ceramics and the tendency of metals to corrode, causing metal ion toxicity. 

Particularly, titanium alloys were used as bone-grafting metals to repair skeletal defects, but insufficient bioactivity was observed [2]. Moreover, permanent implants could still be eroded in vivo, caused by late breakdown and abscess formation [3,4]. In the case of cardiovascular implants, the main drawback is that the materials tend to oxidize and degrade in vivo, creating problems after implantation [5]. Another kind of implants used for the treatment of stress urinary incontinence have disadvantages such as fraying and poor conformity [6].

The addition of polymers to metals or ceramics to form a composite metal-based material could potentially overcome the shortcomings of metals and ceramics for tissue engineering applications [7,8,9,10,11,12]. In this sense biomaterials, including natural polymers such as collagen, gelatin, chitosan and cellulose among others, offer good biocompatibility to be used as implants [13,14,15,16], while synthetic polymers provide good reproducibility and high tenability in the formed composites [7,17].

At this point, it should be taken into account that most of the human body functions including neuronal communication, embryonic development, cardiac beat and damaged tissue repair processes are regulated by electric signals. The materials that interact with active electric tissues are required to be conductive materials so that they can respond to external stimuli and promote adhesion, growth, migration and cellular differentiation [18,19]. In this sense, conjugated polymers are an appropriate alternative for the production of these biomaterials.

Conjugated polymers are recognized as organic materials with similar optical and electric properties to those of certain inorganic materials, including semiconductors or metals. These features are provided by their structure of oxidized polymer and by the negatively charged species that equilibrate their total charge. Polyacetylene, polythiophene, polypyrrole, polyphenylene and polyaniline (PANI) are the most often used conjugated polymers [20]. PANI, in particular, is the polymer with the highest potential because of its easy synthesis, low cost, high electric conductivity and stability. These properties make of it a suitable polymer for different applications, such as biosensors, neuronal catheters, controlled drug release or tissue engineering [21,22]. 

Moreover, the electrical properties of PANI can be adjusted by doping, protonation or charge transfer. This polymer can be used in three different oxidation states according to its conductivity. Emeraldine, the form of PANI that has been used in this study, has a partially oxidized structure, making it a very stable and highly electricity-conductive. Other forms of PANI, such as pernigraniline or leucoemeraldine, which are respectively fully oxidized and reduced forms, present low conductivity, even after doping [20]. 

However, the main drawbacks of using this kind of polymers are their limited mechanical properties as well as their handling and processing difficulties. They are brittle, rigid, barely soluble, easily delaminable, and consequently show low durability [20]. Their combination with other non-conductive synthetic polymers such as poly(lactic-co-glycolic acid) (PLGA), polyvinyl acetate (PVA), polyvinylpyrrolidone (PVP) or polycaprolactone (PCL), among others [22,23,24] has been proposed as a method to overcome such inconveniences and create composites. Moreover, their chemical, electrical and physical properties could be modified to the specific needs of their application by incorporating bioactive substances [25].

In this sense, the production of PANI composites blending it with a non-conductive polymer have been the focus of many investigations. A PANI and polycaprolactone composite has been created for cardiac tissue regeneration [26]. Gil-Castell et al. fabricated nanofibers based on polycaprolactone (PCL) and gelatin with different ratios and polyaniline particles by electro-spinning, thus promoting a controlled increase of the electrical conductivity, and in vitro cardiomyocyte proliferation [27]. In the same way, Rajzer et al. developed PCL/hydroxyapatite scaffolds modified with polyaniline in order to prove that this material enables the growth and proliferation of bone cells [24].

It was also found that the prepared PANI-PMMA composites has a high degree of adhesion and good compatibility with eukaryotic cells [28]. To improve the mechanical properties Gizdavic-Nikolaidis et al. created chitosan polyaniline composites by a rapid method based on microwave-enhanced synthesis that showed greater bactericidal efficacy against Gram-negative *Escherichia coli* and Gram-positive *Staphylococcus aureus* bacteria [29]. Composites of PANI and polypropylene have also been created for neurobiological applications [30].

The polymers used for tissue engineering are required to emulate the extracellular matrices that exhibit the interconnected and homogenous porosity, permeability and mechanical resistance similar to those of in vivo tissue [31,32]. Therapeutic scaffolds must fulfill these requirements. These structures were initially used as just a supportive element in tissue regeneration treatments but at present, they are also employed as a matrix where certain bioactive substances such as anti-inflammatory, antibiotic, antioxidant or even cell-growth stimulating substances are impregnated to be delivered in a controlled manner to favor the regeneration of damaged tissue [33]. 

Most scaffold-synthesizing methods involve the polymerization of a monomer in a solution containing an oxidant and one or more non-conductive polymers. Then, the mixture is poured into moulds to be frozen or dried. However, this technique presents some serious shortcomings, such as the difficulty to fully remove the organic solvent, the high processing temperatures required, which may damage the active substance, or the long processing time, which may result in the stratification of the active substance inside the scaffold and the subsequent reduction of its therapeutic efficiency [34,35,36].

Moreover, the polymeric matrices that can be produced by these conventional methods do not present some of the required morphological characteristics, such as pore size uniformity and the desirable high degree of pore interconnectivity [37]. 

Supercritical CO_2_ foaming is an alternative production method that overcomes these drawbacks and successfully generates functional scaffolds. The high diffusivity and density as well as the low viscosity of the supercritical fluid favors its penetration into the solid matrix and the subsequent dissolution of different active substances. In supercritical foaming processes, the CO_2_ is injected between polymer chains, which results in the plasticization of the polymer and reduces its glass transition temperature. Then, as the system is driven to a supersaturated state, a phase separation takes place that results in the generation of a porous structure inside the polymeric matrix, i.e., a scaffold. This technique requires the use of a polymer with affinity for CO_2_, which explains why it is generally applied to amorphous polymers [38].

Several research works have investigated the use of supercritical CO_2_ as the blowing agent to produce polymeric structures to be used as medical scaffolds. Thus, Cabezas et al. used supercritical foaming to produce biodegradable scaffolds of PLA/PLGA impregnated with indomethacin. In their study, it was concluded that the best properties were achieved when high stirring rates and low depressurization rates were used. The final structures were also more brittle as the PLA ratio was increased [39]. Fanovich et al. [40] generated PCL scaffolds impregnated with natural lichen compounds to be applied in tissue engineering using a one-step extraction-impregnation-foaming process. Scaffolds presenting up to 70% porosity were achieved at 17 MPa and 35 °C. These scaffolds were then impregnated with calcium hydroxyapatite and presented resistance against *Staphylococcus aureus* strains.

Other researchers directly obtained PCL scaffolds for patches of nimesulide by using supercritical CO_2_. In that investigation, the pressure, temperature and contact time were optimized to generate, through one-step processes, scaffolds with a regular porous structure impregnated with the bioactive compounds [41]. Recently, Godoy-Gallardo et al. produced PCL scaffolds impregnated with hydroxyapatite and incorporating the growth factors BMP2 and VEGF to optimize their osteogenic capacity. They reported that the release kinetics data of the growth factors were similar to those observed in in vivo tests [42]. Santos Rosales et al. used supercritical CO_2_ as a foaming and sterilizing agent, developing a one-step process for the production of vancomycin-loaded poly(ε-caprolactone) bone scaffolds. They proved the scaffolds supported the attachment and growth of human mesenchymal stem cells and the biocompatibility, safety and vascularization of the scaffolds [43]. Xiang et al. fabricated a strong, tough poly(lactic acid) foam by combining pressure induced-flow processing with supercritical CO_2_ foaming to obtain biomedical materials with better mechanical properties [44]. All of these results emphasize how important it is to develop a method to produce polymeric scaffolds structures that can be successfully used in tissue engineering.

PCL is a semi crystalline biodegradable aliphatic polyester with good mechanical and thermal properties [41,45]. It is miscible with other polymers such as polyvinyl chloride, polystyrene acrylonitrile, poly(acrylonitrile butadiene styrene), polybisphenol A and other polycarbonates, and it is mechanically compatible with others such as polypropylene, polyethylene or rubber. Moreover, its biodegradability varies according to its molecular weight and crystallinity and may last for periods that range from months to years [46]. 

The aim of this work was to generate systems formed by conjugated (PANI) and non-conductive polymers (PCL) that can be successfully used in tissue regeneration treatments. For this purpose, the authors have evaluated the effects from the main process variables, i.e., temperature (T), pressure (P) depressurization rate (Dr) and PANI/PCL ratio on the polymer expansion factor, its biodegradability, conductivity, mechanical resistance and the textural properties of the final scaffolds.

## 2. Materials and Methods

### 2.1. Materials

Commercial polycaprolactone (PCL) and polyaniline (PANI) emeraldine salt were provided by Sigma–Aldrich (Madrid, Spain). Sodium chloride (NaCl), potassium chloride (KCl), disodium hydrogen phosphate (Na_2_HPO_4_) and potassium dihydrogen phosphate (KH_2_PO_4_) were purchased from Panreac Applychem (Barcelona, Spain). CO_2_ (99.8% minimum purity) was supplied by Linde (Barcelona, Spain).

### 2.2. Supercritical Foaming Process

**Figure 1:**
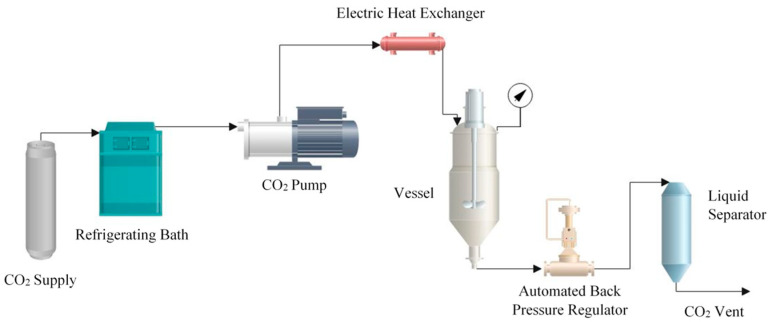
Diagram of the SSI pilot plant used for the experiments.

The foaming processes were carried out in a SSI pilot plant developed by Iberfluid Instrument S.A. (Barcelona, Spain) whose flow diagram is shown in Figure 1. 

This equipment includes a high-pressure pump to fill the foaming vessel with CO_2_ up to the required pressure. The CO_2_ was cooled down by means of an electric heat exchanger fitted with a temperature controller so that it remained in its liquid state and could be pumped into the vessel. The system also includes a 500 mL foaming vessel; a 500 mL cyclonic separator that separates the CO_2_ from the solubilized compounds; a backpressure regulator to control the system pressure; and a micrometric valve to adjust the depressurization rate. 

The experiments were carried out as follows: initially, the corresponding weight of each polymer was placed into an aluminum foil cylindrical support inside the vessel. Then the CO_2_ was pumped into the foaming chamber at the corresponding pressure and temperature. A specific contact time of 1 h was allowed to favor the plasticization of the polymers and then, the CO_2_ was released through the micrometric valve to produce the porous polymer structure. The whole system was controlled by SCADA using an application developed by Iberfluid Instrument S.A.

A set of preliminary experiments were run by varying the PCL/PANI ratio in order to determine the optimal ratio required to obtain a conductive polymer with the minimum amount of PANI residues, i.e., PANI that did not incorporate into the scaffold (runs 1 to 4). Thus, the optimal PCL/PANI ratio was established at 5:1. Then, different pressure, temperature and depressurization rate values were assayed as can be seen in Table 1 (runs 5 to 10).

### 2.3. Scanning Electron Microscopy

A FEI Nova NanoSEM 450^TM^ scanning electron microscope (Thermo Fisher, Frankfurt, Germany) with an accelerating voltage of 30 kV was employed to examine the morphology of the scaffolds. Previously to their analysis, the samples were coated with a 10 nm thick gold layer in order to improve their conductivity. Crossed sections of each polymer were selected for their visualization. 

### 2.4. Porosity Estimation

*Porosity* is defined as the ratio between total void volume (pores) and scaffold total volume. This parameter is calculated based on fluid movement and the Archimedes principle [47,48]. Ethanol (96%) was used as the fluid, since it can penetrate through the polymeric structure without altering it. For the measurement, a scaled tube was filled with a determined volume of ethanol (*V*1). Then the sample was immersed until saturation and the volume was registered (*V*2). Then, the sample was removed from the tube and the ethanol residual volume was noted down as *V*3. Thus, an estimate of the scaffold porosity was achieved according to Equation (1):

(1) $$\;\;\;\;\;\;Porosity\;\left(\%\right)=\frac{volume\;of\;holes}{total\;volume\;of\;scaffold}=\frac{V1-V3}{V2-V3}\cdot 100\;\;\;\;\;\;\;\;\;\;\;\;\;\;\;\;$$

where:

Total volume of Scaffold=(V2−V1)+(V1+V3)=V2−V3

Volume of scaffold=V2−V1

Volume of hole fraction=V1−V3

### 2.5. Estimating the Expansion Factor

The foaming expansion of the scaffold volume was quantified by duplicate [48]. This parameter is defined as the ratio between the *initial* and the *final volumes* (Equation (2)):

(2) $$\;\;\;\;\;\;\;\;\;\;\;\;\;\;\;\;\;Expansion\;Factor=\frac{Final\;Volume}{Initial\;Volume}\;\;\;\;\;\;\;\;\;\;\;\;\;\;\;\;\;\;\;\;\;\;\;\;\;$$

### 2.6. Estimating the Amount of PANI Incorporated into the Scaffold

The amount of *PANI* incorporated into the scaffold was estimated according to the following Equation (3):

(3) $$\;\;\;\;\;\;\;PANI\;load\;\left(\%\;wt\right)=\frac{weight\;of\;PANI\;into\;scaffold}{weight\;of\;initial\;PANI}\times 100\;\;\;\;\;\;\;\;\;\;\;\;\;\;\;$$

The amounts of PCL and *PANI* were initially weighted before they were placed into the foaming vessel. Once the supercritical foaming process was completed, the scaffold was weighted. Thus the difference between the initial PCL weight and the scaffold’s final weight would represent the *PANI* loading into the scaffold. This procedure was conducted in duplicate using different amounts of both polymers.

### 2.7. Biodegradability

A study on the degradation of the scaffolds was conducted by submerging scaffold samples into a 0.01 M pH 7.4 phosphate buffer saline (PBS) solution. First of all, three replicates of each scaffold were dried in an oven at 50 °C for 1 h. Then, these samples were weighted and noted down as *W*1. Then, the samples were submerged into the PBS solution at 37 °C steady temperature. A number of samples were extracted after different time periods (7, 14 and 21 days) and placed on filter paper for 2 h to remove the excess PBS from the scaffold. Then, they were weighed and the weights registered as *W*2 [47]. The *weight loss* of each scaffold sample was calculated according to the following Equation (4):

(4) $$\;\;\;\;\;\;\;\;\;\;\;\;\;\;\;\;\;\;\;Weight\;loss\;\left(\%\right)=\frac{W1-W2}{W1}\times 100\;\;\;\;\;\;\;\;\;\;\;\;\;\;\;\;\;\;\;\;\;\;\;\;\;\;\;$$

### 2.8. Compression Test

The mechanical resistances of the scaffolds were determined according to the Young’s modulus (E) as the slope of the elastic region of the stress-strain curve in a compression test [49]. The compression tests were carried out by means of an Criterion C45 tester (MTS, Frankfurt, Germany) on 15 mm^3^ scaffold cubes. The scaffold cubes were compressed to a total 60% strain at a compression speed of 0.01 mm/s and under 10 kN maximum charge.

### 2.9. Electrical Properties

Each scaffold’s conductivity was indirectly determined by calculating its minimum impedance versus its frequency curves. Impedance is defined as the opposition to electrical flow under a specific voltage, thus, the lower the impedance, the higher the conductivity. The assays were carried out by means of a model 1260 impedance spectroscope (Solartron, Farnborough, UK). 15 mm^3^ cube scaffold samples were subjected at room temperature to 1 V alternating current at frequencies from 1.00 Hz to 1.00 kHz.

## 3. Results and Discussion

### Foaming Process

**Figure 2:**
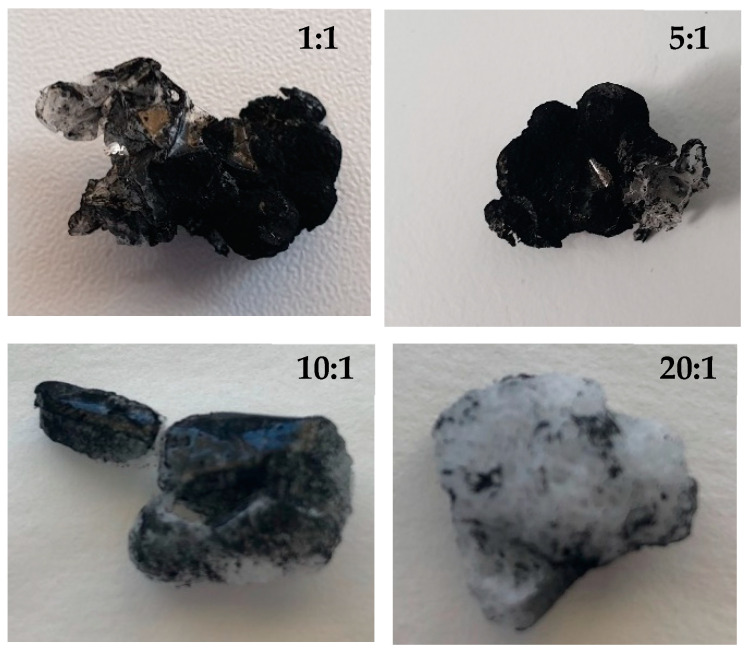
Images of the scaffolds formed in runs 1 to 4.

In the preliminary experiments with PCL and PANI, a large amount of PANI was detected outside the scaffold. Therefore, the PCL:PANI ratio was optimized to avoid this large excess of PANI. The ratio was, consequently, adjusted from 1:1 to 20:1 (runs 1–4) while the rest of the operating conditions remained invariable, as can be seen in Table 1. It was observed that PANI was regularly distributed on the outside surface of the scaffold and also that, as expected, the higher the ratio (larger amount of PANI) the darker the color of the scaffold (Figure 2).

**Figure 3:**
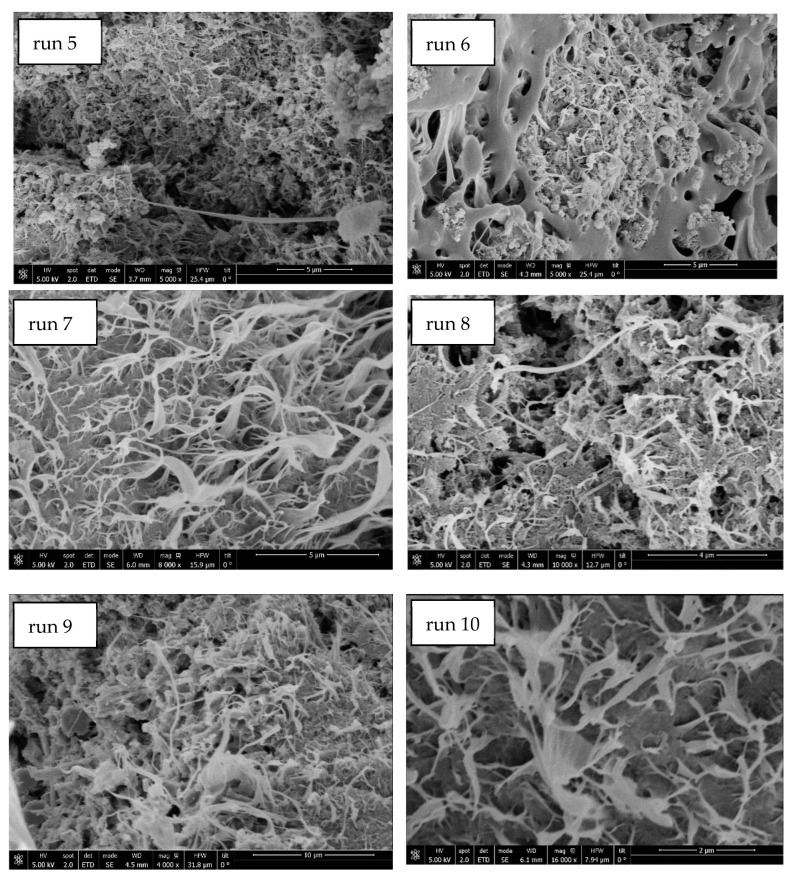
SEM images of the PCL:PANI scaffolds produced by runs 5 to 10.

Moreover, at a 1:1 ratio a greater amount of PANI was found outside the scaffold and part of the PANI that was found on its surface was easily detached. According to the conductivity measured by means of a digital multimeter in a preliminary way, the scaffolds from runs 3 (10:1 PCL:PANI ratio) and 4 (20:1 PCL:PANI ratio) were not electricity conductors, while the ones from runs 1 (1:1 PCL:PANI ratio) and 2 (5:1 PCL:PANI ratio) had a similar conductivity level. Consequently, the scaffolds from ratios 10:1 and 20:1 were discarded and the one from ratio 5:1, where the excess PANI was limited and a suitable conductivity level was registered, was chosen for further experiments. According to the SEM images of the resulting scaffolds that can be seen in Figure 3, the foaming was apparently and successfully completed in runs 5 through 10. 

**Figure 4:**
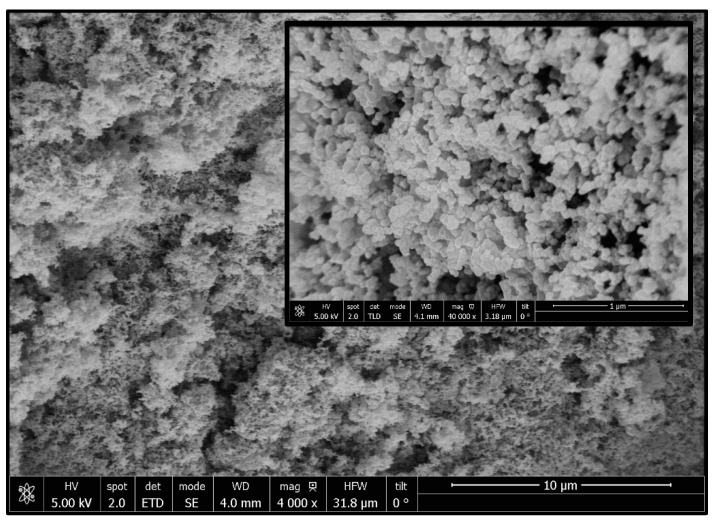
SEM image of a scaffold produced by run 2. A zoomed image is included at the top right corner.

According to the SEM images, the PANI was located inside the cells and on the scaffold’s surface, which represented an inconvenient when trying to visualize the whole scaffold structure. In this sense, the scaffolds were actually formed by foamed PCL, while the PANI was present as spherical particles that had not been foamed. This fact can be clearly observed in Figure 4, where the spherical nanoparticles of PANI on the scaffold’s surface are clearly visible.

**Figure 5:**
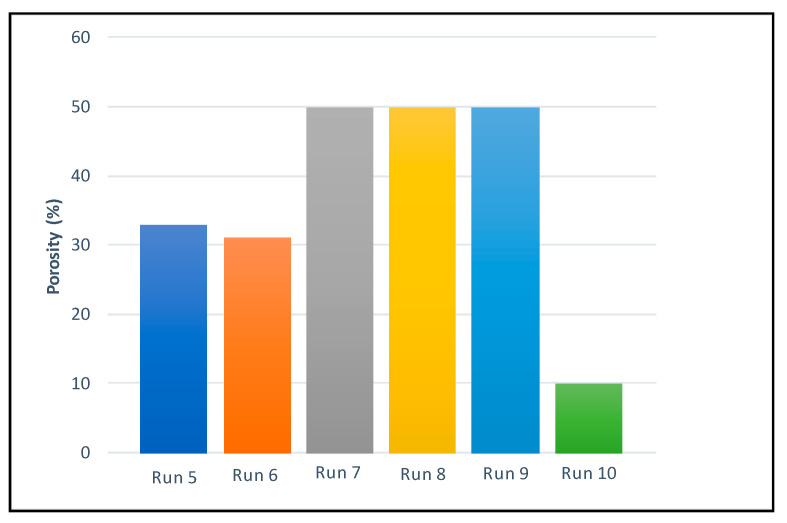
Porosity percentages of the final scaffolds.

Porosity is a crucial factor for scaffolds intended to be used for tissue regeneration. In this work, porosity was calculated based on the Archimedes’ principle, since pores are not easily visualized in SEM images. The porosity values can be seen in Figure 5 and Table 2.

Higher porosity was detected in the scaffolds from runs 7–9, followed by those from runs 5–6. The scaffolds from run 10 presented the lowest porosity. In general, higher temperatures (runs 7–9) seemed to increase the scaffolds’ porosity, while the effect of pressure on the porosity level was neglected, since the scaffolds from runs 7 and 8, which had been produced under the same conditions except for a different pressure level (300 and 100 bar respectively), presented similar porosity, as can be seen in Figure 5. In this sense, Chen et al. [50] found out that the PCL reached its highest porosity (over 80%) when the temperature was raised over 40 °C, or when pressure was increase up to 20 MPa. These conditions seemed to properly promote the melting of the polymer and the dissolution of the CO_2_ and consequently to achieve a successful foaming process [47,50]. In our case this behavior could be affected by the incorporation of PANI.

Other authors have reported that the porosity decreases when the depressurization rate is increased from 18 bar/min up to 75 bar/min [50], while this trend would revert as the depressurization went over 75 bar/min and the scaffolds presented a substantial increment in porosity. Given that in our study only two pressure levels were tested (20 and 50 bar/min) and no significant changes were registered, the effect from depressurization rate on the porosity of the scaffolds was neglected. 

**Figure 6:**
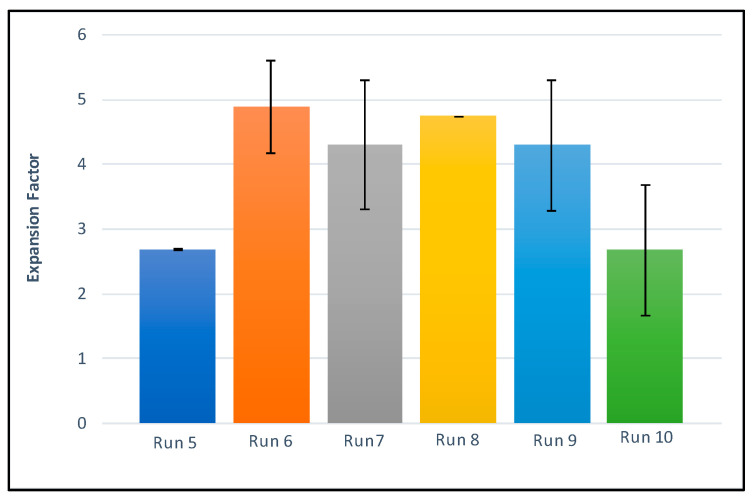
Expansion factor of the scaffolds.

The expansion factor represents the variation of total volume experienced by the polymer structure over the supercritical foaming process. Figure 6 and Table 2 show the data corresponding to this response variable. 

The expansion factor is directly related to porosity, so that a greater expansion factor is expected to be associated to a higher porosity. This is generally in agreement with our results, as can be observed in Figure 4 and Figure 5. Thus, the scaffolds that were formed at the highest temperature tested (above the polymers’ melting temperature, runs 6 to 9) presented a higher expansion factor than those formed at lower temperatures (runs 5 and 10) regardless of the depressurization rate. In fact, the scaffolds from runs 5 and 10, with a very similarly low expansion factor, had been produced at the same low temperature and only the depressurization rate had varied. It could be, therefore, be concluded that, unlike the temperature level, the depressurization rate had no relevant influence on the expansion factor of the final scaffolds at the tested conditions.

**Figure 7:**
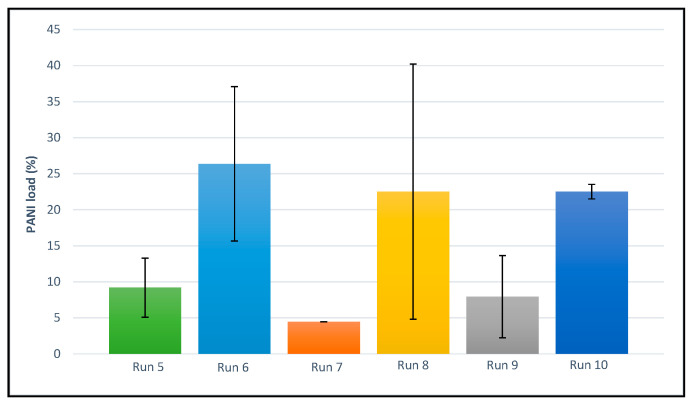
PANI loads incorporated into the structure of the final scaffolds.

The PANI load incorporated into the scaffolds is a crucial factor with regard to their conductivity and, therefore, to determine its suitability for tissue engineering purposes, since its capacity to transmit electrical signals affects communications, growth and cellular adhesion. As can be observed in Figure 7, the scaffold from run 9 exhibited the highest PANI load followed by those obtained from runs 5, 6 and 8. Considerably lower PANI loads were incorporated into the scaffolds produced by runs 5 and 7. It should be noted that runs 5, 6, 8 and 9 were produced at temperatures above the polymer’s melting point and this fact would favor the distribution of the PANI throughout the scaffolds’ structures. Nevertheless, the scaffold produced by run 7 at a lower temperature also presented a relatively large PANI load. In that case, the PANI would probably be located on the surface of the PCL polymer scaffold, since the PANI could not easily penetrate into the structure of an unmelted polymer. This fact could imply a poorer distribution into the structure and consequently a lower conductivity level. 

On the other hand, the scaffolds from run 8 presented a higher dispersion value of the PANI load, which is indicative of its uneven distribution. This run was conducted at a low depressurization rate. In this sense, a low nucleation, as a consequence of such low depressurization rate, produces more irregular foams, since growth is not restricted [50]. In general, according to our results, higher depressurization rates led to greater PANI loads. In fact, fast depressurization led to a higher level of supersaturation by CO_2_, which favors nucleation rather than growth. This would result in the formation of smaller pores where the PANI can get easily trapped and hence the greater PANI loads [50].

The scaffolds’ conductivity was measured indirectly by impedance spectroscopy in order to corroborate that the incorporated PANI provided the scaffolds with electricity conductive properties. As can be seen in Table 2, the lowest impedance levels and, therefore, the highest conductivity was detected in the scaffolds from runs 8 and 9. These runs were conducted at low pressure and high temperature and produced scaffolds with considerable PANI loads. On the other hand, runs 7 and 10 presented the lowest conductivity and varying PANI loads. The scaffolds from runs 5 and 6 exhibited medium conductivity values although they were close to those corresponding to the scaffolds from runs 7 and 10. In all these cases, the PANI loads were quite variable, but their conductivity was similar. This could be due to the uneven distribution of the PANI loads, which did not favor or even prevented electricity flow in some cases. This fact led us to conclude that electrical conductivity did not only depend on the amount of PANI incorporated into the scaffolds, but also on the distribution pattern of the PANI throughout the foam. 

**Figure 8:**
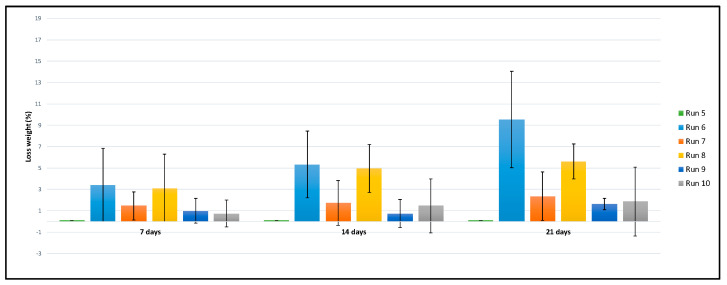
Weight losses of the final scaffolds.

Another factor to be considered is the equilibrium between bio stability and biodegradability, which is crucial to determine the suitability of the scaffolds for tissue engineering purposes. Weight losses of the polymeric structures as considered to indicate polymer degradation. According to the data displayed in Figure 8 and Table 2, the scaffolds generated through run 6 presented the greatest biodegradation, with a final weight loss of 9.54 wt.%, followed by the scaffolds from run 8. On the other hand, the scaffolds from run 5 did not experience any weight variations and therefore, no biodegradation was registered in the assayed time. 

The rest of the scaffolds presented similar biodegradation levels, with weight losses around 1–1.5 wt.% According to the data obtained, most of the scaffolds had a low or slow biodegradability (runs 5, 7, 9 and 10), although the scaffolds from runs 6, with nearly 10 wt.% and run 8 at around 5 wt.%, presented higher biodegradation rates for the tested periods. These experiments were carried out at 70 °C at different pressure and depressurization rate. Although the scaffolds from runs 6 and 8 presented similar PANI loads as well as expansion factor, the scaffolds from run 8 exhibited a higher porosity ratio that would explain their biodegradability differences, since the higher the porosity the higher the biodegradability of the scaffold in the PBS solution [47]. However, the scaffolds from runs 7 and 9, both with a high porosity ratio but different PANI loads, exhibited a low biodegradability. Other authors had already pointed out the difficulty to establish a clear trend due to the interactions that take place between several factors in this type of essays. 

**Figure 9:**
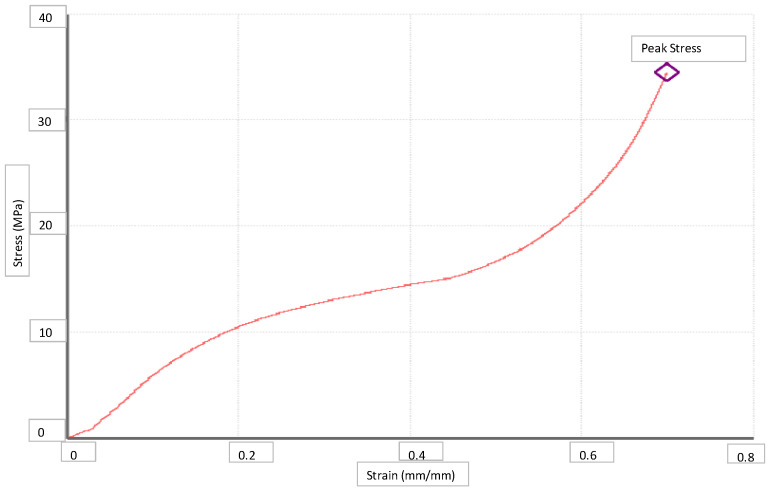
Stress-strain curve of the scaffolds from run 5.

The mechanical resistance of the scaffolds was evaluated in order to determine whether they could be used as cell-supporting matrices for tissue regeneration. Such resistance varies significantly with the type of tissue, ranging between 2 and 6000 MPa depending on whether it is a fibrous or a mature bone tissue, respectively [47,51]. The compression test data were plotted as stress-strain curves. Figure 9 shows the data from the tests on the scaffolds from run 5 as an example. This curve exhibits a lineal region that corresponds to the elastic behavior of the material. The slope of such linear region is the Young’s modulus or linear elastic modulus, which indicates the elastic behavior of the material. Young’s modulus and maximum breaking stress are shown in Table 2. 

The Young’s modulus of the scaffolds from run 6 could not be measured, since it did not exhibit the elastic region that is normally associated to plastic scaffolds and presented irreversible strain under any stress. The scaffolds from run 5 exhibited the highest Young’s modulus, so they were confirmed to have the best mechanical properties. The scaffolds from runs 8 and 10 exhibited medium resistance, while those from runs 7 and 9 presented lower ones. It could be generally said that lower expansion factors and porosity ratios (runs 5 and 8) were directly associated to higher mechanical resistance levels. These last experiments were carried out at higher pressures and lower temperatures. No clear mechanical resistance improvement trends could be associated to variations in the rest of the operating conditions. For an easier comparison, all the data corresponding to each run and their resulting scaffolds have been presented in Table 2 above.

## 4. Conclusions

Polycaprolactone and polyaniline scaffolds for medical usage have been produced by using supercritical CO_2_ as the blowing agent. In general, the scaffolds’ structure was based on foamed polycaprolactone that had incorporated spherical polyaniline particles inside the scaffold’s pores or on its surface. The influence from specific process variables, namely pressure, temperature, depressurization rate and PCL/PANI ratio on the response variables, namely expansion factor, porosity ratio, polyaniline load, biodegradability, mechanical resistance and conductivity were determined. A 5:1 PCL:PANI ratio was established to evaluate the rest of the process parameters, since at this ratio, PANI residues were limited and the resulting scaffolds presented satisfactory conductivity properties. In general, the scaffolds that had been formed at high temperature (above the PCL melting temperature) presented a higher expansion factor and porosity ratio that those formed at low temperatures. The effect of pressure and depressurization rate on both response parameters were not conclusive.

Greater polyaniline loads were achieved at higher temperatures, where the melted polycaprolactone would facilitate a more thorough distribution of the load inside the scaffolds’ structure. On the other hand, the polyaniline loads would be located on the surface of the polymer foam when the experiments were conducted below polycaprolactone’s melting temperature, since the polyaniline particles could not easily penetrate the foam structure. In general, and according to the data from the essays that have been completed, a higher depressurization rate results in greater polyaniline loads. In this sense, the scaffolds from the experiments that achieved greater loads of polyaniline, which had been conducted at lower pressure and higher temperature, exhibited higher conductivity. This is a crucial factor, since these matrices have to interact with active electric tissues and only those materials with the adequate conductive properties can successfully respond to external stimuli and promote the necessary adhesion, growth, migration and cellular differentiation.

On the other hand, in general the higher porosity, the higher biodegradability by the PBS solution. Also, higher mechanical resistance was exhibited by the scaffolds with a low expansion factor and a low porosity ratio.

In any case and due to a number of exceptions to some of the tentative trends that have been observed, a number of undetermined interactions that take place over the foaming process do not allow for a precise trend to be established. Anyway, when operating conditions of 70 °C, 100 bar, a ratio of 5:1 (PCL:PANI), a depressurization rate of 20 bar/min and a contact time of 1 h were used, a scaffold with high porosity and expansion factor, high PANI load and conductivity and moderate mechanical properties and biodegradability was achieved.

## Figures and Tables

**Table 1:** Supercritical foaming experiments.

Runs	P (bar)	T (°C)	Dr (bar/min)	Ratio PCL:PANI
1	300	40	20	1:1
2	300	40	20	5:1
3	300	40	20	10:1
4	100	40	20	20:1
5	300	40	50	5:1
6	300	70	50	5:1
7	300	70	20	5:1
8	100	70	20	5:1
9	100	70	50	5:1
10	300	40	20	5:1

**Table 2:** Summary of data corresponding to runs and their resulting scaffolds.

Run	P (bar)	T (°C)	Dr (bar/min)	P ^1^ (%)	EF ^2^ (%)	PL ^3^ (%)	B ^4^ (%)	I ^5^ (Ω)	E ^6^ (MPa)	PS ^7^ (MPa)
5	300	40	50	33	2.68	9.19	1.64	1.90·10^8^	58.24	34.51
6	300	70	50	31	4.88	28.50	2.35	2.03·10^8^	-	8.47
7	300	70	20	50	4.30	4.46	1.87	4.67·10^8^	2.56	8.02
8	100	70	20	50	4.74	22.51	5.59	2.35·10^5^	12.00	3.61
9	100	70	50	50	4.29	7.93	9.54	1.99·10^6^	2.41	1.76
10	300	40	20	10	2.67	22.48	0.00	4.85·10^8^	15.56	10.41

^1^ P = Porosity; ^2^ EF = Expansion Factor; ^3^ PL = Polyaniline load; ^4^ B = Biodegradability; ^5^ I = Impedance; ^6^ E = Young Modulus; ^7^ PS = Peak Stress.

## Data Availability

The data presented in this study are available on request from the corresponding author.
